# First-Line Durvalumab Plus Platinum-Etoposide *Versus* Platinum-Etoposide for Extensive-Stage Small-Cell Lung Cancer: A Cost-Effectiveness Analysis

**DOI:** 10.3389/fonc.2020.602185

**Published:** 2020-12-04

**Authors:** Longfeng Zhang, Yongfu Hang, Maobai Liu, Na Li, Hongfu Cai

**Affiliations:** ^1^Department of Thoracic Oncology, Fujian Provincial Cancer Hospital, Fujian Medical University Cancer Hospital, Fujian Medical University, Fuzhou, China; ^2^Department of Pharmacy, The First Affiliated Hospital of Soochow University, Suzhou, China; ^3^Department of Pharmacy, Fujian Medical University Union Hospital, Fujian Medical University, Fuzhou, China

**Keywords:** durvalumab, platinum–etoposide, cost-effectiveness, extensive-stage small-cell lung cancer, small cell lung cancer

## Abstract

**Background:**

The aim of the present study was to evaluate the cost-effectiveness of durvalumab plus platinum–etoposide *versus* platinum–etoposide as first-line treatments for small-cell lung cancer from the perspective of the US payer.

**Methods:**

This study established a partition survival model for three health states, metastasis probability, and safety data based on the CASPIAN clinical trial. The health utility value was mainly derived from the published literature. Only direct medical costs were considered. Sensitivity analyses were conducted to assess the robustness of the incremental cost per quality-adjusted life year (QALY).

**Results:**

Durvalumab plus platinum–etoposide increased QALY by 0.220 compared to that observed with platinum–etoposide only. The cost increased by $78,198.75 and the incremental cost per QALY increased by $355,448.86. One-way and probability sensitivity analyses indicated that the model parameters varied within a limited range and had no significant effect on the results.

**Conclusions:**

Although durvalumab plus platinum–etoposide can improve quality of life, it also substantially increases the cost of medical treatment. Under a willingness-to-pay threshold of $100,000, durvalumab does not have a cost-effective comparative advantage.

## Introduction

Lung cancer is a malignant tumor associated with high levels of morbidity and mortality worldwide (1). Small cell lung cancer (SCLC) accounts for 13–17% of all lung cancers (2). SCLC is characterized by a high degree of malignancy, a short doubling-time during tumor cell multiplication, widespread metastasis over a relatively short period of time at early stages of diagnosis, and a poor prognosis. The 5-year survival rate is only 1–5% (3). In approximately two-thirds of SCLC cases, the cancer progresses and exhibits extensive-stage at the time of initial diagnosis (4, 5). The treatment of extensive-stage SCLC (ES-SCLC) is mainly based on radiotherapy and chemotherapy. Most patients with SCLC are sensitive to the first-line standard treatment of etoposide combined with platinum-based dual drugs. Nevertheless, almost all patients with SCLC will inevitably develop drug resistance and tumor recurrence with an objective response rate (ORR) of 50–60% (6).

Immunotherapy using immune checkpoint inhibitors (ICIs) has revolutionized cancer therapy and has provided relief to patients with SCLC (7, 8). Durvalumab, a selective, high-affinity humanized monoclonal antibody binds to programmed death-ligand 1 (PD-L1; CD274) and blocks its interactions with programmed cell death protein 1 (PD-1) and CD80 (B7.1) (9). The CASPIAN study, a randomized, open, multi-center, phase III clinical trial of durvalumab involving the first-line treatment of patients with ES-SCLC, explored the efficacy and safety of durvalumab plus platinum–etoposide *versus* platinum–etoposide alone. The overall survival (OS) in the durvalumab combination chemotherapy group was increased to 13.0 months, compared with 10.3 months in the chemotherapy alone group (HR = 0.73; p = 0.0047). Moreover, durvalumab combined with chemotherapy reduced the risk of cancer-associated death by 27%. The investigator-assessed confirmed ORR was 68% (95% CI: 62–73%) in the durvalumab plus chemotherapy arm and 58% (95% CI: 52–63%) in the chemotherapy only arm (10). On the basis of the results of the Phase III CASPIAN study, the US Food and Drug Administration (FDA) approved the combination of durvalumab with etoposide, carboplatin, or cisplatin, as a first-line treatment for patients with ES-SCLC in March 2020 (11).

The present study aimed to assess the cost-effectiveness of durvalumab plus platinum–etoposide *versus* platinum–etoposide as a first-line treatment for patients with ES-SCLC from the perspective of the US payer.

## Materials and Methods

A partitioned survival model was developed to simulate the clinical outcome and economic cost of two first-line strategies, namely (1) immunotherapy and (2) chemotherapy. The immunotherapy groups received up to four doses of durvalumab plus platinum–etoposide, followed by maintenance of durvalumab every 4 weeks. The chemotherapy group received up to six doses of platinum–etoposide and preventive intracranial irradiation. Patients presenting disease progression or unacceptable adverse reactions were treated with a second-line treatment. The specific drugs of the second-line treatment plan were not specified in the original RCT study (including appendix). A small proportion of the patients received subsequent immunotherapy (2% in the durvalumab plus platinum–etoposide group; 5% in the platinum–etoposide group) (10). In agreement with both the recommendations of the National Comprehensive Cancer Network (NCCN) guidelines and with the recommendations of the relevant random phase 3 trial, topotecan was provided to all patients as a post-progress option (12, 13).

**Figure 1:**
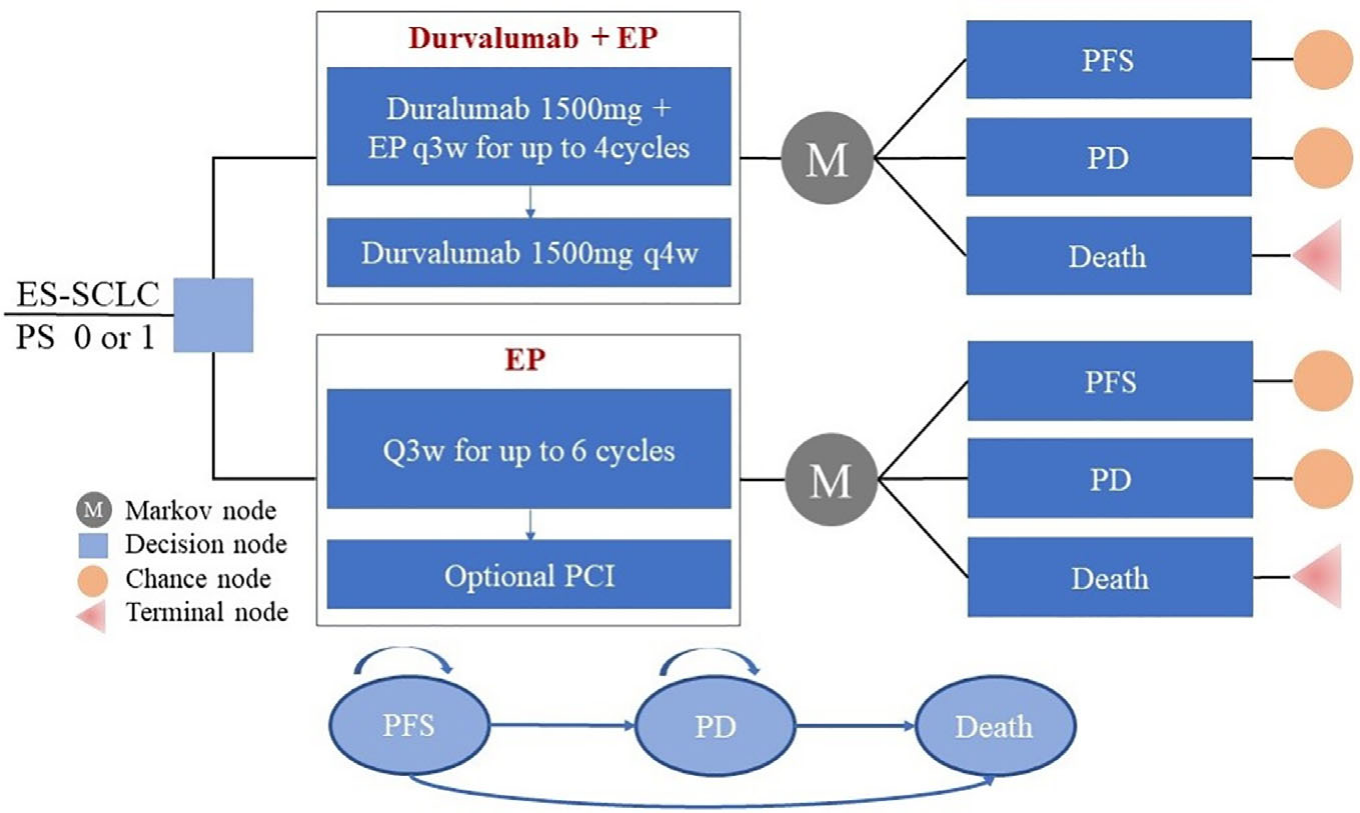
Partitioned survival model simulating outcomes for the CASPIAN trial. The model considers the transition states of ES-SCLC. All patients start in the PFS state and receive treatment with the two treatment plans. Patients can enter the state of PD and subsequently move to the state of the death. EP, platinum-etoposide; PFS, progression-free survival; PD, progressed disease; PCI, prophylactic intracranial irradiation; ES-SCLC, extensive-stage small-cell lung cancer.

Our model structure included the following three states: progression-free survival (PFS); progressed disease (PD); and death (**Figure 1**). All patients were considered to be in a state of PFS at the time of enrolment. During each cycle of simulation, patients were assigned to a certain state, received the corresponding treatment, incurred a specific treatment cost, and gained a certain health effect. The cycle of the model was set to 3 weeks in line with the clinical research and lung cancer treatment pathways. Based on a consideration of the average age of patients in the CASPIAN study (62 years of age) and of expected overall survival time, the time duration was set to 10 years (a period expected to include the patient’s entire life span). Only direct medical costs were considered, and a 3% discount rate for health utility and cost was assumed (14).

The primary outcomes of our model simulation included total cost, quality-adjusted life years, and life years (LY). The incremental cost-effectiveness ratio (ICER) was also calculated and compared with a willingness-to-pay (WTP) threshold of $100,000 per QALY; calculated from a cost-effectiveness analysis on the basis of the recommendations of Neumann et al (15). If the ICER was less than or equal to the threshold, then the intervention plan was more economical than the control plan. If the ICER was greater than the threshold, then the control plan was more economical than the intervention plan. Basic and sensitivity analyses were conducted using a model constructed with the TreeAge Pro 2018 software (TreeAge, Williamstown, Massachusetts).

### Transition Probabilities

Clinical efficacy data for the first-line treatments, including the Kaplan–Meier (K–M) curves of PFS and overall survival (OS), were derived from the Phase III CASPIAN study (10). The GetData Graph Digitizer (version 2.26; http://www.getdata-graph-digitizer.com/download.php) was used to extract the PFS and OS probabilities from the PFS and OS curves of each treatment group (16). The individual patient data of each K–M curve was re-constructed and survival analysis was used to fit the data according to the method of Hoyle et al (17). The long-term clinical outcome survival function was obtained from fitting and extrapolation of the K–M curve. Distribution functions considered included exponential, Weibull, log-normal, gamma, and log-logistic (18). Akaike information criterion (AIC) and Bayesian information criterion (BIC) were used to test the goodness of fit; low AIC and BIC values indicated better fit (19). The goodness of fit of different distributions of OS and PFS curve data is shown in **Supplementary Table A.1**. The Weibull distribution function provided the best fit of the PFS and OS data. Lambda (*λ*) and gamma (*γ*) were calculated using the K–M curve simulation and extrapolation according to the survival function of the Weibull distribution S(t) = exp(−*λ*t*^γ^*) (20). The probability of PFS to death in the model was assumed to be natural mortality (21). PFS and OS derived from the model simulation were compared with the clinical trial data to provide an internal validation of our model (**Supplementary Figure A.1**). The median PFS and OS estimates derived from the model were acceptably close to those presented in the CASPIAN trial (**Supplementary Table A.2**).

### Health Utilities

The health utility values of PFS and PD health status were derived from published studies. The utility values of PFS and PD in the two groups were 0.673 and 0.473, respectively (22–24). The product of the adverse reaction utility value and the incidence rate were used to calculate the utility loss caused by each adverse reaction. Adverse reactions were considered only at grade 3 or 4. Significant differences were observed between the two groups regarding specific adverse reactions (neutropenia and anemia). While the incidences of neutropenia and anemia in the immunotherapy plus chemotherapy groups were 24 and 9%, respectively, the incidences in the chemotherapy only group were 33 and 18%, respectively (10).

### Cost Estimates

Direct medical costs included drug procurement, administration, and the cost of treating adverse events (25). In our study, durvalumab was recommended for four cycles at a dose of 1,500 mg every 21 days, followed by continued use of durvalumab every 4 weeks until progression. We assumed that a one-cycle dose of the chemotherapy drug included etoposide 90 mg/m^2^, carboplatin area under the curve (AUC) of 5 mg/ml/min, cisplatin 80 mg/m^2^, and topotecan 1.5 mg/m^2^/d (10, 26). Patients in the immunotherapy group received four cycles of chemotherapy. Patients in the chemotherapy group were allowed six cycles of chemotherapy. With regard to the clinical trial population treated with cisplatin and carboplatin, 75% of the patients were treated with carboplatin. Approximately 8% of the patients received one dose of prophylactic intracranial irradiation (PCI) in the chemotherapy group (10). Sensitivity analyses were performed for the probability and dose range to avoid the influence of parameters on the results. Based on the median age of inclusion in the CASPIAN trial, initial model patients had the following characteristics: age, 62 years; average body weight, 70 kg; surface area, 1.8 m^2^; and creatinine clearance rate, 70 ml/min (27, 28). Our model assumed no drug wastage. We calculated the unit price of each medicine on the basis of Medicare part B drug average sales price from the U.S. Centers for Medicare & Medicaid (29).

The administration and radiotherapy costs were calculated according to the Medicare physician fee schedule for 2020 (25, 30, 31). The durations of chemotherapy drug and durvalumab single-drug infusion were 3 and 1 h, respectively. Therefore, while each cycle of the chemotherapy group required 3 h, each cycle of the immunotherapy group required 4 h in total. The total drug administration cost per patient was defined as the product of the unit cost of drug administration for each chemotherapy regimen multiplied by the mean number of cycles (28, 32).

The costs for patient follow-up visits and chemotherapy administration are shown in **Table 1**. The radiotherapy costs are summarized in **Supplementary Table A.3**. The cost of grade 3 or grade 4 adverse reactions was derived from previously published articles (33, 34); only the adverse reactions showing differences were included.

**Table 1:** Model parameters.

Variable	Baseline value	Range		Reference
		Minimum	Maximum	
Weibull PFS survival model				
Durvalumab plus EP	*λ* = 0.060321292, *γ* = 1.33964421	–	–	(10)
EP	*λ* = 0.033423371, *γ* = 1.87592327	–	–	(10)
Weibull OS survival model				
Durvalumab plus EP	*λ* = 0.022633982, *γ* = 1.35549307	–	–	(10)
EP	*λ* = 0.017262037, *γ* = 1.56573969	–	–	(10)
Durvalumab plus EP: Incidence of AEs				
Neutropenia	0.24	0.19	0.29	(10)
Anemia	0.09	0.072	0.11	(10)
EP: Incidence of AEs				
Neutropenia	0.33	0.26	0.40	(10)
Anemia	0.18	0.14	0.22	(10)
Utility				
PFS	0.673	0.27	0.8	(22–24)
PD	0.473	0.19	0.56	(22–24)
Neutropenia	−0.46	−0.368	−0.552	(22)
Anemia	−0.073	−0.058	−0.088	(22)
Drug cost per mg, US $				
Durvalumab	7.6179	6.0943	9.1415	(29)
Etoposide	0.0602	0.04816	0.07224	(29)
Carboplatin	0.05714	0.04571	0.06857	(29)
Cisplatin	0.1845	0.1476	0.2214	(29)
Topotecan	12.11	9.688	14.532	(29)
Drug administration and routine management cost, US $				
Outpatient follow-up visit	52.33	41.86	62.80	(25, 30)
Administration IV, first hour	142.22	113.78	170.66	(25, 30)
Administration IV, additional hour	30.68	24.54	36.82	(25, 30)
AEs cost, US $				
Neutropenia	17,181	13,745	20,617	(33)
Anemia	20,260	16,208	24,312	(33)

PFS, progression-free survival; EP, platinum–etoposide; OS, overall survival; AE, adverse event; PD, progressed disease; IV, intravenous.

### Sensitivity Analysis

To assess stability, one-way sensitivity analysis was used to analyze the influence of different parameter changes on the results. The variation ranges of the related parameters in the univariate analysis were derived from published studies, 95% confidence intervals, or ±20% variations. The results are presented in a tornado diagram. Second-order Monte Carlo simulations were also used for probabilistic sensitivity analyses. Based on the recommendations of the ISPOR-SMDM Modeling Good Research Practice Working Group, the incidence parameters were set to beta distribution, the cost and medical resource utilization parameters were set to gamma distribution, and the health utility value parameters were set to beta distribution (35). The probabilistic sensitivity analyses were repeated 1,000 times, and the ICER of the two treatments was calculated each time. The results are presented using a cost-effectiveness acceptability curve.

## Results

### Base Case Results

The results of our basic cost-effectiveness analysis are shown in **Table 1**. In the durvalumab plus platinum-etoposide group, the cumulative life years of patients was 0.991 years, and the cumulative QALY was 0.550 QALY. These estimates were 0.417 years (cumulative life years) and 0.220 QALY (cumulative QALY) higher than the corresponding values in the platinum–etoposide group. The total cost per patient in the durvalumab plus platinum–etoposide group was $90,072.83, which was $78,198.75 higher than the per patient cost in the platinum–etoposide group. Overall, durvalumab plus platinum–etoposide as a first-line treatment for ES-SCLC had an ICER of $355,448.86 per QALY compared to lone treatment with platinum–etoposide (**Table 2**). Thus, durvalumab plus platinum–etoposide had an ICER value higher than the willingness-to-pay threshold (this is considered a non-cost-effective advantage). If durvalumab was only administered seven times in total, then the total cost of durvalumab plus platinum-etoposide was $75,332.74, and the total utility was 0.55 QALY. These estimates were $63,458.66 and 0.22 QALY higher than those of the control group. In this instance, the ICER was $288,448.45 per QALY, which was markedly higher than the threshold.

**Table 2:** Cost-effectiveness analysis.

Strategies	Life years	QALYs	Total costs(US$)	ICER(US$/QALY)(Durvalumab plus EP versus EP)
Durvalumab plus EP	0.99	0.55	90,072.83	355,448.86
EP	0.57	0.33	11,874.08	
Incremental (Durvalumab plus EP versus EP)	0.42	0.22	78,198.75	

EP, platinum–etoposide; QALYs, quality-adjusted life-years; ICER, incremental cost-effectiveness ratio.

### Sensitivity Analysis

**Figure 2:**
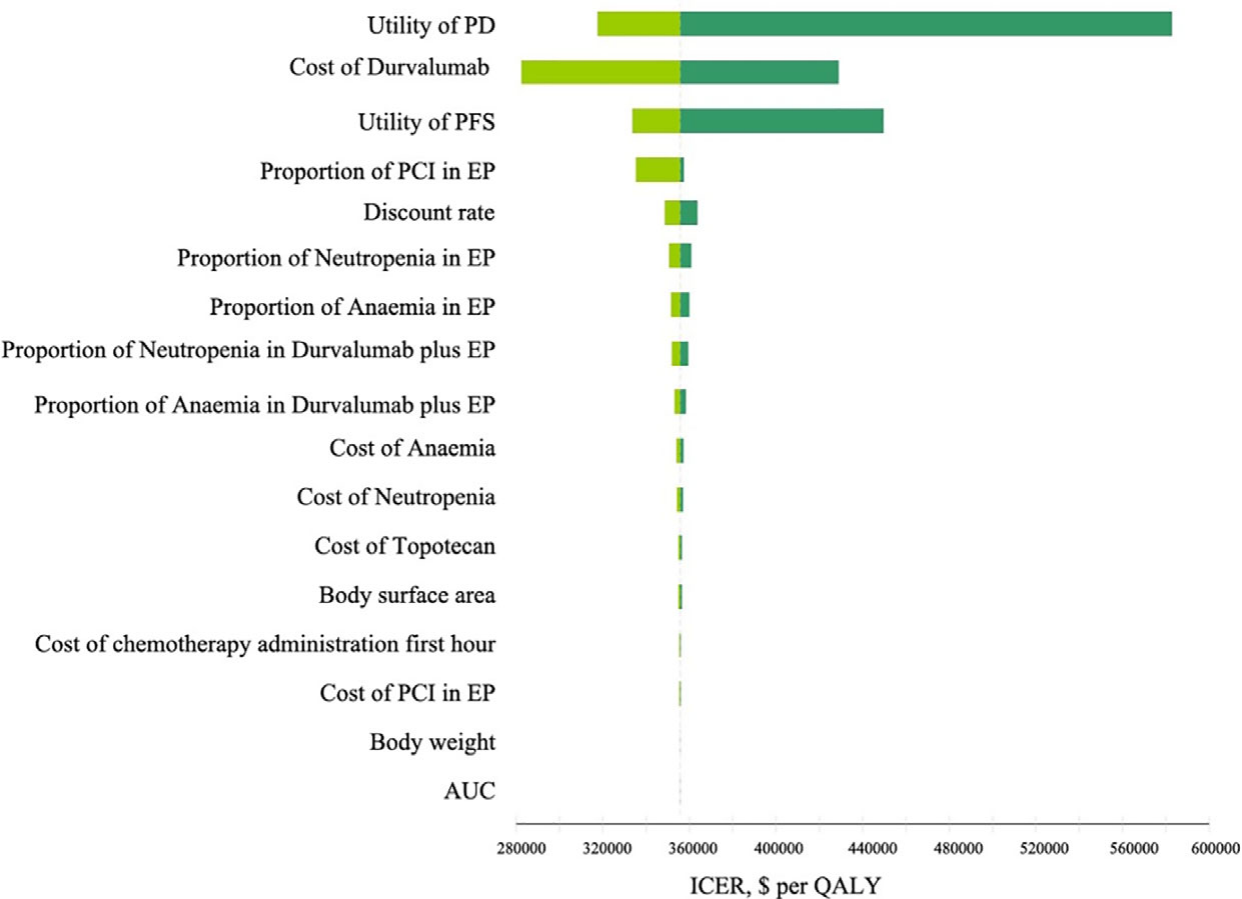
Tornado diagram of one-way sensitivity analyses comparing the first-line durvalumab plus platinum-etoposide versus platinum-etoposide for extensive-stage small-cell lung cancer. The dotted line intersecting the light and dark green bars represents the ICER of $355,448.86 per QALY from the base case results. PD, progress disease; PFS, progression-free survival; EP, platinum-etoposide; PCI, prophylactic intracranial irradiation; AUC, area under curve; ICER, incremental cost-effectiveness ratio; QALY, quality-adjusted life years.

The results of the one-way sensitivity analysis are illustrated in **Figure 2**. The utility of PD, the cost of durvalumab, and the utility of PFS were the main influencing factors on ICER. Several additional parameters, including the proportion of patients undergoing PCI in the chemotherapy group, the proportion of cisplatin and carboplatin chemotherapy, body weight, AUC, and body surface area, had a slight effect on ICER.

**Figure 3:**
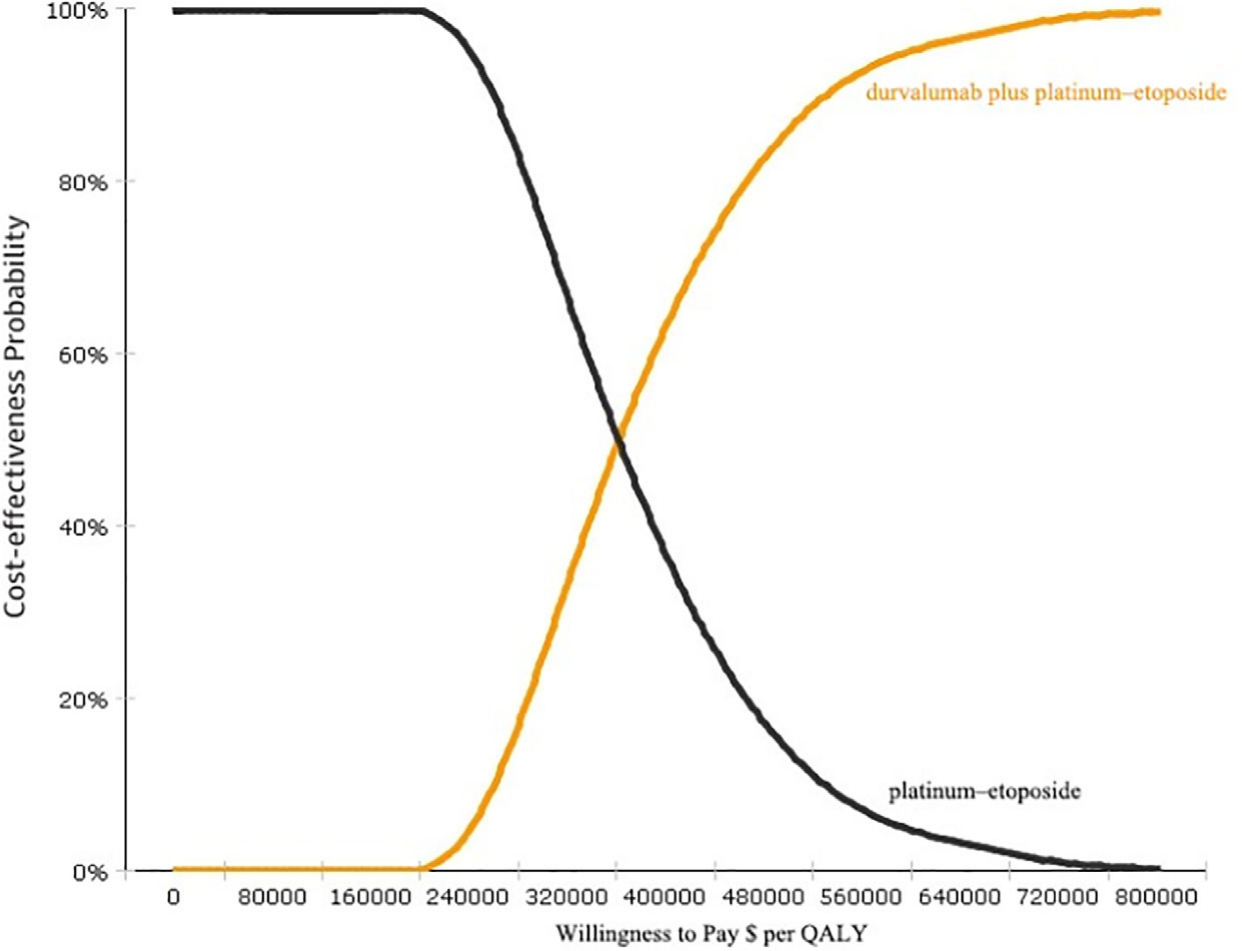
Cost-effectiveness acceptability curves (CEAC). CEAC is a curve used to indicate the probability of a drug being economical. The magnitude of the WTP value directly affects the cost-effectiveness of the protocol. The acceptable curve shows the percentage of the cost-effectiveness of the simulation by using different treatment options.

The results of the probabilistic sensitivity analyses are shown in **Figure 3**. The cost-effectiveness acceptability curve demonstrated that the probability of durvalumab immunotherapy with a cost-effectiveness advantage was 0% under a threshold of $100,000. If the threshold was reduced to $360,000, then the probability that durvalumab had a cost-effectiveness advantage was 51.2%. Thus, the probability of a cost-effectiveness advantage of durvalumab is likely to increase with an increase in the average social willingness to pay.

## Discussion

Treatment options for SCLC are limited. Two independent large-scale phase III clinical studies, CASPIAN and IMpower133, have provided evidence that immunotherapy plus chemotherapy can extend the overall survival of patients with ES-SCLC (8, 10). Based on a consideration of the results of these clinical studies, the FDA approved atezolizumab or durvalumab (the PD-L1 inhibitors) combined with chemotherapy for the first-line treatment of ES-SCLC. According to the CASPIAN study, durvalumab plus platinum–etoposide significantly improved the OS of patients compared with standard chemotherapy. The reported safety profile was also consistent with that reported in previous studies involving durvalumab and platinum–etoposide (10). Thus, durvalumab is the new drug of choice for ES-SCLC patients. However, while immunotherapy has achieved encouraging results in clinical studies, durvalumab is relatively expensive. Therefore, it remains to be determined whether the price of durvalumab reflects the clinical value of the drug, whether the medical insurance system will underwrite the costs of the drug, and whether patients will ultimately be treated with the drug.

According to our study, durvalumab combined with platinum-etoposide for ES-SCLC is not cost-effective from the perspective of the US payer. The incremental cost per patient is $78,198.75. While the incremental QALY is 0.220, the ICER is $355,448.86 per QALY. The utility and cost of durvalumab are two factors that have a considerable effect on our model. However, no utility value data has been published for ES-SCLC patients. In accordance with the approach used by Zhou et al., the utility values in PFS and PD states refer to NSCLC patients (23). To avoid the utility value from disproportionately affecting the stability of the results, we referred to multiple studies to expand the range of utility values in the sensitivity analyses. The results indicate that the utility value does not have a substantial effect on the results. The model simulation also indicated that if the cost of durvalumab was reduced by 55%, then ICER was close to 150,000 per QALY. If the cost of durvalumab was reduced by 70%, then ICER was close to 100,000 per QALY. Therefore, in order for the durvalumab scheme to have a cost-effective advantage over chemotherapy, the price of durvalumab must be reduced more than 70% (to achieve the WTP threshold of $100,000 per QALY). If the threshold is raised to 150,000, then the price should be reduced by more than half of the current price.

Our study is the first to evaluate the economics of durvalumab combined with chemotherapy for ES-SCLC through economic modeling methods and the latest evidence. To date, many studies have been conducted on the cost-effectiveness of durvalumab in the treatment of NSCLC. Steven et al. have provided evidence that durvalumab consolidation therapy is more cost-effective than no consolidation therapy after chemoradiotherapy in patients with unresectable stage III NSCLC in the US healthcare system (36). Han et al. have reported that first-line durvalumab consolidation therapy can be cost effective (compared with placebo) for patients with unresectable stage III NSCLC from the US payers’ perspective (37). However, the conclusions of these studies differ from the conclusions obtained in our study. In the above-mentioned studies, durvalumab may be economical because the survival time of NSCLC patients is significantly longer than that of patients with SCLC. Thus, although the drugs are expensive, long-term treatment demonstrates good clinical benefits. The economics of immunotherapy for SCLC have only been reported in one previous study. Zhou et al. compared the cost-effectiveness of atezolizumab plus carboplatin and etoposide with the standard first-line chemotherapy from an American perspective. The cost of atezolizumab treatment increased overall cost by $52,881 compared with chemotherapy, and ICER was $528,810 per QALY, with an increase in 0.1 QALYs (23). The authors concluded that immunotherapy combined with chemotherapy was not a cost-effective approach, which was consistent with the results of our study.

The optimal duration of ICIs remains unknown (38). Indeed, evidence has been presented to suggest that an increase in the dose of immune checkpoint inhibitors (ICIs) does not necessarily lead to long OS. Moreover, patients who discontinue ICI due to toxicity or other reasons may continue to show a benefit. Presently, few clinical studies have been conducted on the combination of chemotherapy and immunotherapy in the first-line treatment of SCLC (7). Patients in the IMpower133 and CASPIAN trials were treated with ICI until disease progression or unacceptable toxicity. In clinical studies, the median number of durvalumab doses received during first-line treatment was seven; 24% of the patients received 12 doses or more. In our model, we determined the cost and utility of first-line durvalumab for seven doses, for one year, two years, or lifetime, to avoid the influence of the duration of durvalumab on the results. Our results indicate that the durvalumab plus chemotherapy regimen is not economical in all situations.

This study has several limitations. First, to our knowledge, the CASPIAN trial is the only randomized phase III trial that has compared durvalumab plus platinum-etoposide with platinum-etoposide in ES-SCLC (10). This is a large and well-designed trial, but our model is intrinsically dependent on the validity and generalizability of the trial, and any bias within the trial will be reflected in our research. Owing to the strict selection and exclusion criteria, patients entering clinical trials are generally younger and healthier. As several clinical trials are in progress, the current model may need to be updated upon further publication of new data. Second, outcomes outside the observation period of the CASPIAN trial were obtained by fitting the parameter distribution to the K–M curve and by extrapolating the PFS and OS data. Although our model has been verified, this may increase uncertainty in the model output. Third, the cost analysis does not include the cost of grade 1 and 2 adverse events. However, these adverse reactions were relatively minor, with less remarkable effects on cost. Fourth, different chemotherapy regimens may be administered after disease progression. For simplification of the model, the choice of chemotherapy drugs was not considered. Fifth, we used Medicare reimbursement to estimate the drug costs in the model. Although Medicare reimbursement may be lower than commercial reimbursement, the lack of publicly available sources reporting commercial drug prices poses challenges in the usage of commercial reimbursement for cost-effectiveness analyses.

Despite the above-mentioned limitations, none of the model parameters seem to disproportionately affect the findings. Indeed, sensitivity analysis indicates that probability, utility, and cost data are unlikely to affect the final result. The results of this study reflect the general clinical treatment of patients with ES-SCLC. Thus, the study has a reference value for doctors and policy makers.

## Conclusion

In the treatment of ES-SCLC, durvalumab is unlikely to be cost-effective for the WTP threshold of $100,000 or $150,000 per QALY. If immunotherapy becomes pivotal in first-line treatment, then the cost of durvalumab needs to be considerably reduced to increase economic efficiency.

## Data Availability Statement

The original contributions presented in the study are included in the article/**Supplementary Material**. Further inquiries can be directed to the corresponding author.

## Author Contributions

LZ: Data curation, investigation, writing—original draft preparation. YH: Data curation, investigation. HC: Conceptualization, methodology, writing, reviewing and editing. NL: Software. ML: Supervision, visualization. All authors contributed to the article and approved the submitted version.

## Funding

The funding agencies had no role in the study design, data collection and analysis, decision to publish, or manuscript preparation. This study was supported by Fujian Provincial Department of Science & Technology (grant no. 2017Y0035) of the People’s Republic of China and the National Natural Science Foundation of China (grant no. 71804025).

## Conflict of Interest

The authors declare that the research was conducted in the absence of any commercial or financial relationships that could be construed as a potential conflict of interest.
