# Supplementary figures and images for: First-Line Durvalumab Plus Platinum-Etoposide Versus Platinum-Etoposide for Extensive-Stage Small-Cell Lung Cancer: A Cost-Effectiveness Analysis
Source: Front Oncol. 2020 Dec 4;10:602185. doi: 10.3389/fonc.2020.602185 (PMC7747765; doi:10.3389/fonc.2020.602185)

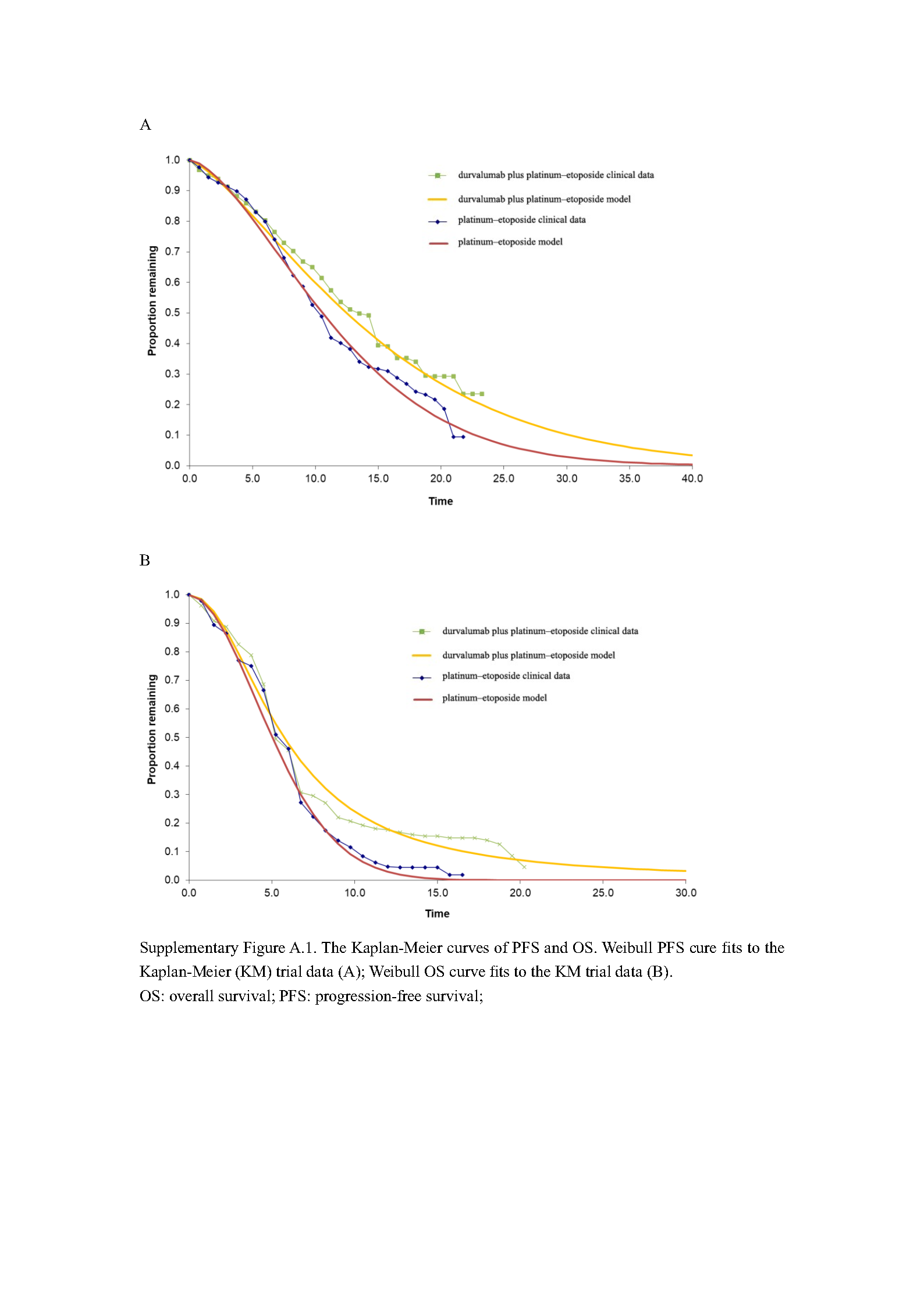

Supplement: Supplementary file 1 [file Image_1.tiff]
