# Supplementary material for: First-Line Durvalumab Plus Platinum-Etoposide Versus Platinum-Etoposide for Extensive-Stage Small-Cell Lung Cancer: A Cost-Effectiveness Analysis
Source: Front Oncol. 2020 Dec 4;10:602185. doi: 10.3389/fonc.2020.602185 (PMC7747765; doi:10.3389/fonc.2020.602185)
Supplement: Supplementary file 2 [file Table_1.docx]

**Supplementary Table A.1. Comparison of survival models**

|  | AIC | | BIC | |
| --- | --- | --- | --- | --- |
|  | Durvalumab  plus EP | EP | Durvalumab  plus EP | EP |
| OS |  |  |  |  |
| Exponential | 1295.80 | 1440.57 | 1299.36 | 1444.17 |
| Weibull | 1280.78 | 1400.85 | 1284.34 | 1404.45 |
| Logistic | 1322.83 | 1439.97 | 1326.39 | 1443.57 |
| Log-normal | 1309.45 | 1434.66 | 1313.00 | 1438.26 |
| Log-logistic | 1292.49 | 1412.35 | 1296.05 | 1415.94 |
| PFS |  |  |  |  |
| Exponential | 1524.69 | 1415.83 | 1528.28 | 1419.39 |
| Weibull | 1483.21 | 1306.24 | 1502.57 | 1309.81 |
| Logistic | 1591.61 | 1324.74 | 1595.20 | 1328.31 |
| Log-normal | 1500.84 | 1359.84 | 1504.43 | 1363.41 |
| Log-logistic | 1498.98 | 1347.36 | 1486.79 | 1350.93 |

AIC: Akaike information criterion; BIC: Bayesian Information Criterion; EP: platinum–etoposide; OS: Overall survival; PFS: Progression-free survival;
