# Supplementary material for: First-Line Durvalumab Plus Platinum-Etoposide Versus Platinum-Etoposide for Extensive-Stage Small-Cell Lung Cancer: A Cost-Effectiveness Analysis
Source: Front Oncol. 2020 Dec 4;10:602185. doi: 10.3389/fonc.2020.602185 (PMC7747765; doi:10.3389/fonc.2020.602185)
Supplement: Supplementary file 3 [file Table_2.docx]

| internal validation of model | model | clinical data |
| --- | --- | --- |
| median OS on durvalumab plus EP | 13.00 months | 12.49 months |
| median PFS on durvalumab plus EP | 6.1 months | 5.1 months |
| median OS on EP | 10.57 months | 10.3 months |
| median PFS on EP | 5.04 months | 5.4 months |
| the 12-month PFS rates on durvalumab plus EP | 18% | 18% |
| the 12-month PFS rates on EP | 3% | 5% |

**Supplementary Table A.2. Internal validation of model**

OS: overall survival; PFS: progression-free survival; EP: platinum–etoposide;
