# Supplementary material for: First-Line Durvalumab Plus Platinum-Etoposide Versus Platinum-Etoposide for Extensive-Stage Small-Cell Lung Cancer: A Cost-Effectiveness Analysis
Source: Front Oncol. 2020 Dec 4;10:602185. doi: 10.3389/fonc.2020.602185 (PMC7747765; doi:10.3389/fonc.2020.602185)
Supplement: Supplementary file 4 [file Table_3.docx]

| Activity | CPT | Description | Unit | Cost($) |
| --- | --- | --- | --- | --- |
| Simulation | 77290 | Set radiation therapy field | 1 | 508.14 |
| Physician planing | 77263 | Radiation therapy planning | 1 | 174.3128 |
| Physics plan | 77307 | Teletherapy, isodose plan, complex | 1 | 295.9347 |
|  | 77334 | Radiation treatment aid(s) | 3 | 130.2835 |
| Treatment | 77280 | Set radiation therapy field | 1 | 283.3034 |
|  | G6003 | Radiation treatment delivery | 10 | 189.8313 |
|  | G6002 | Stereoscopic x-ray guidance | 10 | 76.50995 |
|  | 77336 | Radiation physics consult | 2 | 81.2016 |
| Management | 77427 | Radiation treatment management, per 5-treamtnet | 2 | 196.3274 |
|  |  | Total: $4,871.01 |  |  |

**Supplementary Table A.3. Radiotherapy Costs**
